# Eye Movement Indicator Difference Based on Binocular Color Fusion and Rivalry

**DOI:** 10.3390/jemr18020010

**Published:** 2025-04-05

**Authors:** Xinni Zhang, Mengshi Dai, Feiyan Cheng, Lijun Yun, Zaiqing Chen

**Affiliations:** 1 School of Information Science and Technology, Yunnan Normal University, Kunming 650500, China; 2324100061@ynnu.edu.cn (X.Z.); chengfy03@163.com (F.C.); yunlijun@ynnu.edu.cn (L.Y.); 2 Engineering Research Center of Computer Vision and Intelligent Control Technology, Department of Education of Yunnan Province, Kunming 650500, China; dms18487097603@163.com; 3 Yunnan Key Laboratory of Optoelectronic Information Technology, Kunming 650500, China

**Keywords:** binocular color fusion, binocular color rivalry, eye movement, Z-Score normalization analysis, ROC analysis

## Abstract

Color fusion and rivalry are two key information integration mechanisms in binocular vision, representing the visual system’s processing patterns for consistent and conflicting inputs, respectively. This study hypothesizes that there are quantifiable differences in eye movement indicators under states of binocular color fusion and rivalry, which can be verified through multi-paradigm eye movement experiments. The experiment recruited eighteen subjects with normal vision (nine males and nine females), employing the Gaze Stability paradigm, Straight Curve Eye Hopping paradigm, and Smoothed Eye Movement Tracking paradigm for eye movement tracking. Each paradigm included a binocular color rivalry experimental group (R-G) and two binocular color fusion control groups (R-R, G-G). Data analysis indicates significant differences in indicators such as Average Saccade Amplitude, Median Saccade Amplitude, and SD of Saccade Amplitude between binocular color fusion and rivalry states. For instance, through Z-Score normalization and cross-paradigm merged analysis, specific ranges of these indicators were identified to distinguish between the two states. When the Average Saccade Amplitude falls within the range of −0.905–−0.693, it indicates a state of binocular color rivalry; when the range is 0.608–1.294, it reflects a state of binocular color fusion. Subsequently, ROC curve analysis confirmed the effectiveness of the experimental paradigms in analyzing the mechanisms of binocular color fusion and rivalry, with AUC values of 0.990, 0.741, and 0.967, respectively. These results reveal the potential of eye movement behaviors as biomarkers for the dynamic processing of visual conflicts. This finding provides empirical support for understanding the neural computational models of binocular vision and lays a methodological foundation for developing visual impairment assessment tools based on eye movement features.

## 1. Introduction

When the left and right eyes receive different images, the brain processes these inputs in two distinct ways: slight differences lead to binocular fusion, while significant differences result in binocular rivalry [1,2,3]. Binocular fusion and rivalry represent complementary phenomena in binocular vision that are frequently discussed in studies concerning consciousness, attention, and the brain’s processing of conflicting visual information. Breese revealed the dynamics of inhibition between opposing colors using green-red stimuli [4], a study later expanded by Gellhorn and Schoeppe who explored color dominance with varying intensities [5]. From the 20th century onwards, research shifted from monocular to binocular rivalry, with Levelt proposing models of alternating dominance [6] and Blake and Fox examining the hierarchy of visual processing through aftereffects [7]. Wandell et al. contributed a model explaining how the brain integrates color information from both eyes [8].

Entering the 21st century, Li et al. demonstrated that binocular stimulation training could significantly improve visual integration and perceptual stability in adult amblyopic patients, highlighting the potential of such training for visual rehabilitation [9]. Brascamp et al. reviewed Levelt’s laws of binocular rivalry, revealing that periodicity and stability are closely related to neural oscillatory activity, providing new insights into understanding its dynamic mechanisms [10]. Riesen et al. found that the visual system can flexibly switch between rivalry and fusion, indicating that external stimuli and neural processing influence these perceptions [11]. Chen et al. quantified the effects of inconsistency and color difference on binocular color rivalry, discovering that increased inconsistency enhances rivalry likelihood, whereas minor inconsistencies favor fusion [12]. Lv et al. analyzed EEG signals using the EEGNet model, showing that dorsal areas play a significant role in distinguishing color fusion and rivalry [13]. Asano and Wang introduced a preliminary rivalry indicator ΔE*bino, proving the critical role of color differences in binocular rivalry and offering a new tool for quantifying these processes [14].

Color fusion and rivalry are two key information integration mechanisms in binocular vision, representing the visual system’s processing modes for consistent and conflicting inputs, respectively. Existing studies have shown that binocular color rivalry and fusion phenomena are closely related to the neural mechanisms of visual information processing, involving processes such as attention allocation [15], conflict monitoring [16], and multisensory integration [17]. However, most existing studies focus on macroscopic representations of behavioral responses or EEG signals, with few systematically exploring the potential value of eye movement indicators in distinguishing fusion from rivalry.

This research gap may stem from two aspects: first, traditional experimental designs often view eye movements as nuisance variables, suppressing their natural behavior by fixing gaze points [18], thereby ignoring the function of eye movements as “indicators of cognitive load”; second, the rapid dynamics of rivalry and fusion (such as millisecond-level perceptual switching) impose higher spatiotemporal resolution requirements on eye movement data [19]. However, with advancements in high-precision eye movement technology and machine learning, the potential of eye movement indicators in parsing microsecond-level cognitive processes (such as conflict detection and attention switching) is gradually becoming evident. For instance, Raveendran et al. discovered that saccadic movements can modulate the efficiency of binocular field integration [20], while Kalisvaart and Goossens further proposed that interactions between eye movement signals and additional retinal signals might encode rhythmic characteristics of competitive alternation [21]. These findings suggest that eye movement behaviors are not only passive reflections of perceptual outputs but also actively participate in the dynamic process of conflict resolution.

Notably, although some studies have focused on the role of tiny eye movements in maintaining visual stability [22,23], they have yet to clearly answer a crucial question: Can eye movement indicators effectively distinguish cognitive states of color fusion and rivalry? Hirota et al. found that decreased binocular fusion ability could serve as an indicator of visual fatigue [24], but their study did not address mechanisms regulating color conflicts. Grossberg et al. revealed associations between saccade velocity and depth perception but did not explore specific responses in color rivalry [25]. Furthermore, existing eye movement studies primarily focus on monochrome or grayscale stimuli [26], lacking systematic analysis of the regulatory mechanisms of asymmetric adaptation across color channels [27].

Therefore, this study aims to systematically analyze eye movement indicators under states of binocular color fusion and rivalry through multi-paradigm eye movement experiments, including the Gaze Stability paradigm, Straight Curve Eye Hopping paradigm, and Smoothed Eye Movement Tracking paradigm. We hypothesize that eye movement indicators will show significant differences when distinguishing between states of binocular color fusion and rivalry.

## 2. Methods

### 2.1. Instrumentation and Viewing Groups

**Figure 1:**
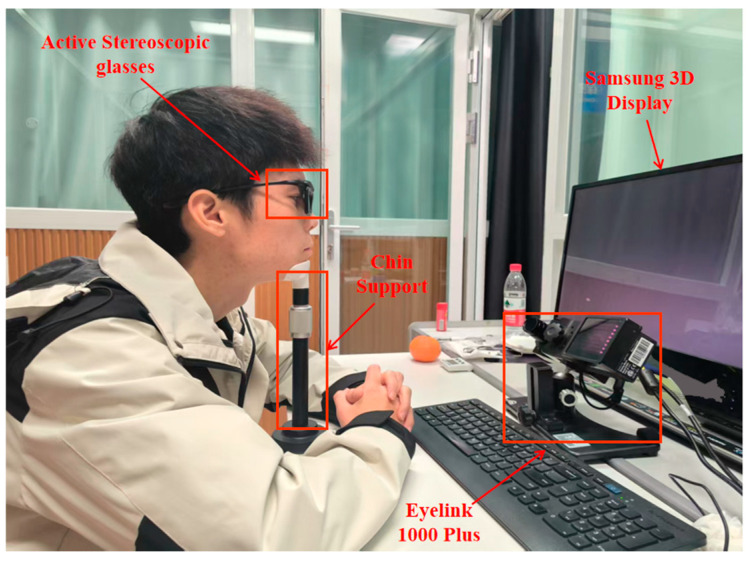
Experimental environment and equipment diagram.

Figure 1 illustrates the apparatus and equipment used in our experiments. Visual stimuli were presented on a 3D display (Samsung Electronics, Suwon, Gyeonggi-do, Republic of Korea) paired with matching 3D shutter glasses(Samsung Electronics, Suwon, Gyeonggi-do, Republic of Korea). The display features a 2D/3D switching capability, with dimensions of 511.8 mm (H) × 288.3 mm (V), a resolution of 1920 × 1080 pixels, and a refresh rate of 120 Hz. The 3D shutter glasses enabled viewing of the 3D stimuli by pressing a switch button. Stimuli were calibrated to specific CIE-1931 coordinates and luminance using the look-up table (LUT) method. Brightness and chromaticity were measured at the display’s center through the glasses using a spectroradiometer (Photo Research PR-715, Photo Research, Granite Bay, CA, USA), achieving a characterization accuracy of 1.56 CIELAB units ($∆E_{ab}^{*}$). A chin support device secured the participant’s head, minimizing deviations due to spontaneous movement. Binocular eye movement data were collected with an EyeLink1000 Plus (SR Research: Ottawa, ON, Canada) video eye tracker at 2 kHz. All experiments were conducted in a dark room, with participants seated 50 cm from the display.

### 2.2. Subjects

Eighteen subjects (nine males and nine females) participated in visual acuity and visual function testing, meeting all experimental criteria. The subjects were aged between 22 and 30 years old, with 11 wearing glasses and 7 not. All subjects had normal visual acuity, no refractive errors, no color blindness or color vision deficiency, and normal stereopsis. Each subject signed an informed consent form and participated in the experiment anonymously [28]. A detailed report on the vision of the 18 subjects is provided in Appendix A Table A1.

In the context of binocular color rivalry, various factors of the visual system, such as visual acuity, can influence attention allocation and rivalry dynamics. Lower visual acuity may lead the visual system to prioritize more salient stimuli, thereby affecting target visibility and information detection efficiency [29]. Therefore, to ensure the reliability of experimental data, we implemented strict screenings of visual acuity and visual function for all participants.

Ophthalmologists, along with trained research assistants (author Xinni Zhang), conducted comprehensive screenings. Visual acuity was evaluated using logarithmic scales at a standard distance of 4 m for both uncorrected and corrected vision. For myopic subjects, specialized optometric instruments were used to quantify corrected visual acuity accurately. Following this, in-depth visual function tests were conducted, including assessing color vision with the Ishihara Color Blindness Test and measuring depth perception using the Titmus Stereoscopic Chart (stereoscopic sensitivity ≤ 100 arcsec). Additionally, the presence and location of the blind spot were determined using a traditional method, where a research assistant guided the participant to track a small pointer while it was moved gradually.

This rigorous screening procedure ensured uniformity among all participants in terms of visual acuity, color vision, and stereoscopic vision, thereby minimizing potential individual differences that could impact the experimental results and enhancing the reproducibility and robustness of this study’s conclusions.

### 2.3. Stimuli Paradigm

In the CIELAB color space, all stimulus colors were selected at a lightness (L*) of 30, with a luminance of 15 ± 0.5 cd/m^2^. Building on previous research [30], we quantified the binocular chromatic fusion limit, establishing that binocular color rivalry occurs when the color difference exceeds 27.55. To elicit a pronounced rivalry effect, we presented two opposing colors—R: L*a* b*(30, 30, 0) and G: L*a* b*(30, −30, 0), referred to as R-G—to each eye separately.

**Figure 2:**
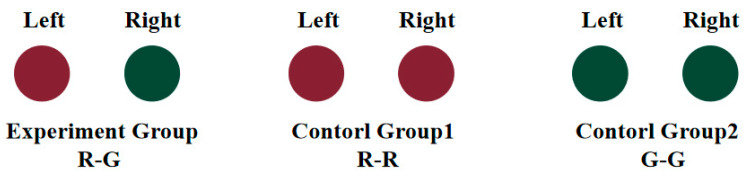
Stimuli paradigm of the experimental and control groups.

Heterogeneous fusion involves the brain perceiving the integration of identical colors from both eyes into a more vivid hue, while homogeneous fusion leads to the perception of mixed or contrasting colors. These experimental groups enable the investigation of binocular fusion mechanisms and the integration of different colors by the visual system. In the heterogeneous fusion condition, color differences can produce complex phenomena, like color mixing and depth perception. Conversely, homogeneous fusion minimizes color interference, allowing for a clearer exploration of the basic fusion mechanisms. Thus, we selected homogeneous stimuli (R-R and G-G) for the control group to facilitate normal binocular color fusion (see Figure 2). To prevent background interference with color fusion effects, the stimuli were presented against a black background with a luminance of 0.24 cd/m^2^.

Three experimental paradigms were utilized: the Gaze Stability paradigm, Straight Curve Eye Hopping paradigm, and Smoothed Eye Movement Tracking paradigm [31,32,33,34]. Each paradigm included an experimental group (R-G) and two control groups (R-R and G-G), with equal presentation times across all groups.

**Figure 3:**
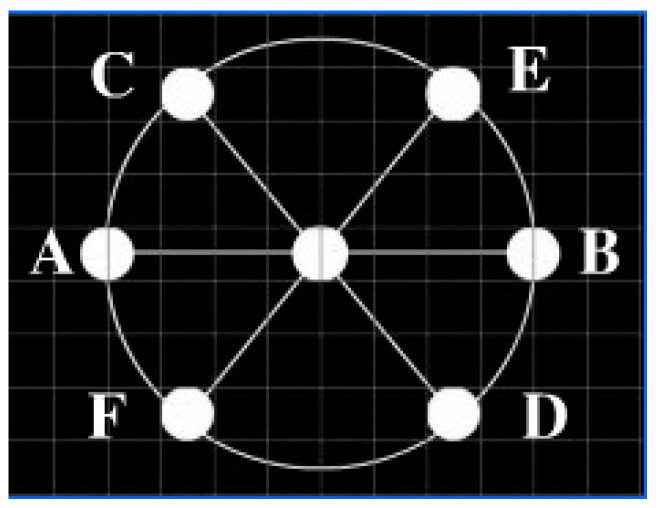
Paradigm of Gaze Stability.

In the Gaze Stability paradigm (see Figure 3), a target disk with a diameter of 2° is fixed at the center of the screen and presented continuously for 10 s. Subsequently, a distractor disk, identical in size and color to the target disk, appears randomly at one of six preset positions (A–F) for 500 ms before disappearing. The distractor disk is presented in random order and cycles through all positions. Participants are required to fixate on the target disk throughout the entire 130 s duration until all positions have been tested.

**Figure 4:**
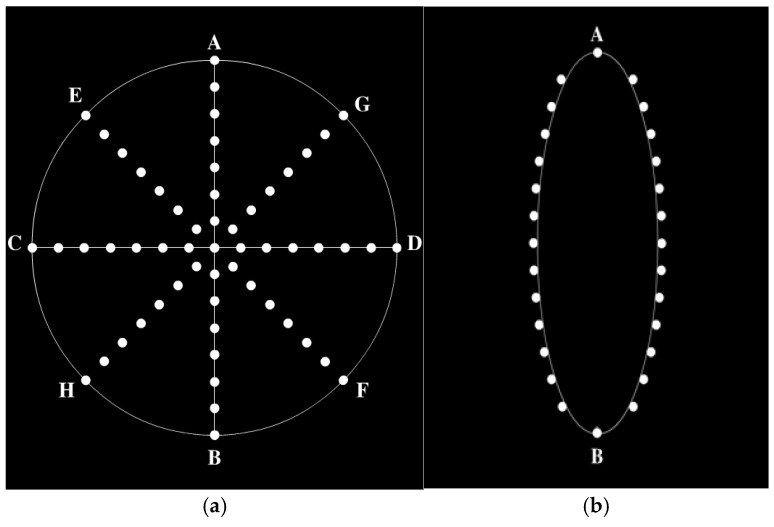
Paradigm of Straight Curve Eye Hopping: (**a**) straight-line path (**b**); curve path.

The Straight Curve Eye Hopping paradigm (see Figure 4) consists of two movement paths: straight and curved. In Figure 4a, the disk moves linearly between points A and G at a speed of 4.5° per 1.2 s for 14 repetitions. In Figure 4b, the disk starts at points A or B and follows two arcs at the same speed. This segment lasts a total of 100 s, with 38 s for the straight path and 62 s for the curved path. Participants were required to track the disk’s movements with their eyes.

**Figure 5:**
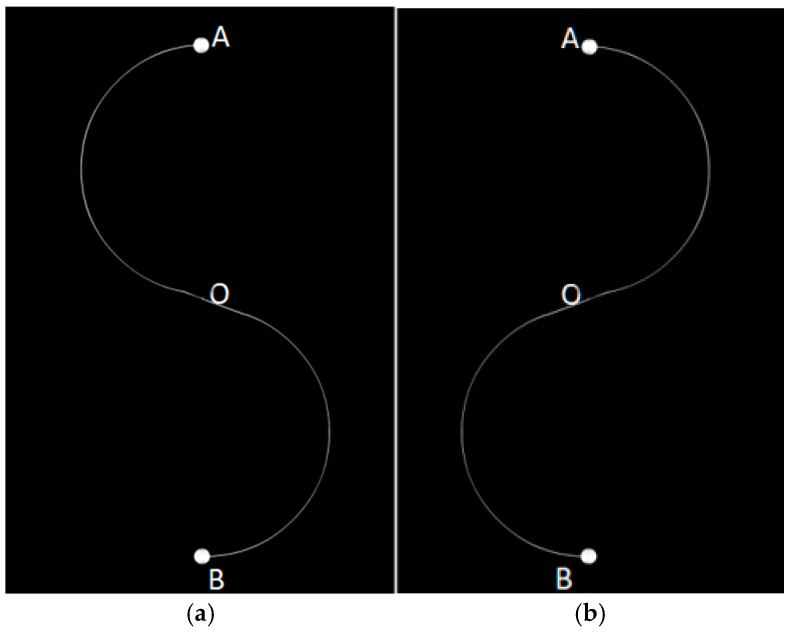
Paradigm of Smoothed Eye Movement Tracking: (**a**) “S”-shaped path; (**b**) reverse “S”-shaped path.

In the Smoothed Eye Movement Tracking paradigm (see Figure 5), the disk moves back and forth along an “S”-shaped path, beginning from points A or B. The entire experiment lasts 80 s, with each “S” and its reverse taking 40 s. Participants were instructed to maintain smooth eye movements to follow the disk’s trajectory.

### 2.4. Procedure

**Figure 6:**
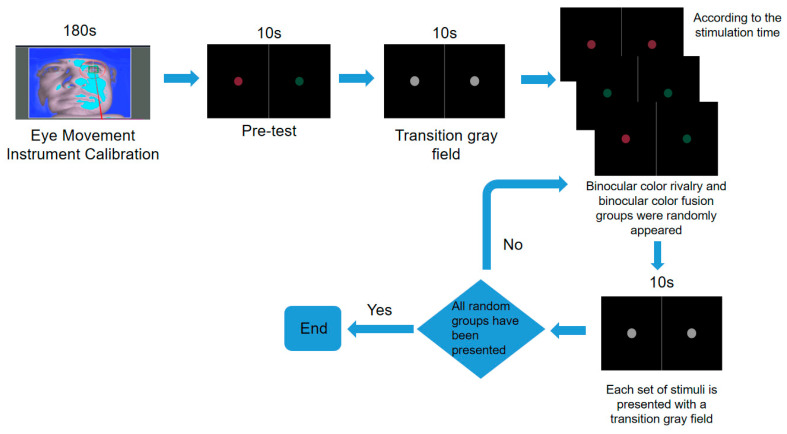
Schematic diagram of one experimental cycle.

The experimental procedure is illustrated in Figure 6. To enhance the alertness and concentration of participants, the experiment was scheduled in the morning, starting at 8:00 and ending at approximately 9:20, with each session lasting about 80 min and only one participant involved per day. Prior to the start of the experiment, participants were required to wear active stereo glasses, adjust their seating, and complete calibration on the eye movement instrument to ensure data accuracy (for detailed calibration steps, see Appendix B). After calibration, a 10 s pre-test was conducted where participants had to report whether they perceived any rivalry phenomena. If perception was reported, the experiment proceeded; otherwise, the experiment was paused.

To minimize measurement errors, a 10 s transitional gray field was displayed before and after each stimulus presentation, ensuring equal exposure time for both eyes to identical stimuli. Stimulus presentations were randomized, with rivalry and fusion stimuli alternating. Throughout the experiment, participants performed eye movement tasks according to the requirements of different paradigms.

The completion of all random groups marked the end of one experimental cycle. Repeating this cycle three times indicated the completion of data collection.

### 2.5. Data Processing and Statistical Analysis

Data processing commenced with the elimination of erroneous records and the exclusion of data from participants who failed to complete tasks as required. Outliers within the eye movement data were identified and removed using box plots alongside the 3σ principle. To ensure comparability across participants, we calculated the Mean and standard deviation (SD) for each eye’s movement data, as well as for both eyes combined, followed by normalization of relevant indicators to mitigate the impact of individual differences on analysis outcomes. Each participant completed three repetitions under both experimental and control states to minimize biases stemming from distractions, instrument misalignment, or calibration errors. The raw data and stimulus materials are accessible via the following link: https://doi.org/10.6084/m9.figshare.25688625.v1 (dataset accessed on 25 April 2024).

To assess significant interocular differences, a paired *t*-test was conducted on the left and right eye data within the same experimental group, with Bonferroni correction applied (adjusted α′ = 0.0013). Given that binocular fusion and rivalry involve the brain’s integration of inputs from both eyes to form a single percept, rather than isolating contributions from each eye, data from both eyes were merged. For binocular eye movement data conforming to a normal distribution, an independent samples *t*-test was employed. Otherwise, a Mann–Whitney U test was used for non-normally distributed data. After detecting indicators with significant differences across paradigms, we further performed a Z-score normalization analysis to summarize the ranges of these indicators, removing overlapping ranges between binocular color fusion and rivalry states to more precisely distinguish based on eye movement features. To ensure that the obtained indicator ranges could be effectively applied in non-controlled experiments, we conducted a cross-paradigm analysis of eye movement indicators, identifying the intersection ranges of indicators that showed significant differences across the Gaze Stability paradigm, Straight Curve Eye Hopping paradigm, and Smoothed Eye Movement Tracking paradigm, thereby highlighting common features among the paradigms. Additionally, ROC analysis was utilized to evaluate the capability of the three paradigms in distinguishing between binocular color fusion and rivalry. All statistical analyses were executed using SPSS 27.0 software, with a significance level set at *p* < 0.05 (corrected where applicable).

## 3. Results

### 3.1. Results of Interocular Significant Difference

Appendix A Table A2, Table A3 and Table A4 summarize the results, showing significant differences in Gaze X, Gaze Y, and Pupil Size, while Acceleration X, Acceleration Y, and Velocity X remained stable across all three paradigms.

Gaze X, Gaze Y, and Pupil Size reflect eye position and pupil response, which are sensitive to shifts in visual input and binocular vision integration. These differences are expected due to changes in visual tasks. In contrast, Acceleration X, Acceleration Y, and Velocity X, which measure the eyes’ speed and acceleration, showed no significant differences, consistent with Robinson’s theory that eye movements are centrally controlled to ensure coordination between both eyes during movement [35].

Since binocular fusion and rivalry reflect the brain’s ability to integrate and process inputs from both eyes into a single percept, we focus on analyzing the averaged data from both eyes to capture the visual system’s collaborative response rather than isolating monocular contributions.

### 3.2. Results of Normal Distribution Analysis

The results of the normal distribution test are listed in Appendix A Table A5, and *t*-tests and Mann–Whitney U tests for all three experimental paradigms are listed in Appendix A Table A6, Table A7, Table A8 and Table A9.

**Figure 7:**
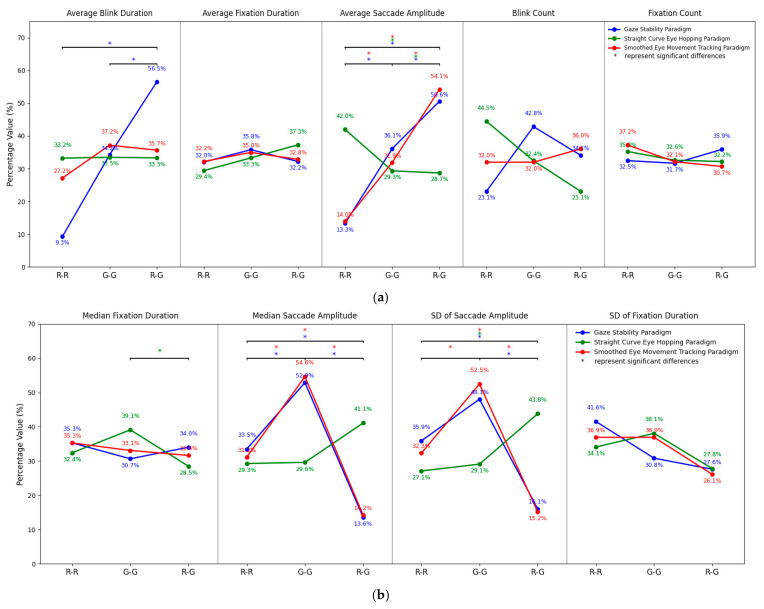

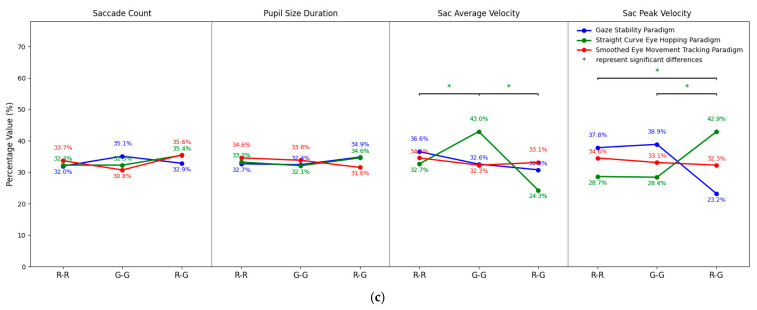
Normalized eye movement data for the R-G, R-R, and G-G groups. (**a**) Graph 1 displays the percentage values of eye movement indicators; (**b**) Graph 2 displays the percentage values of eye movement indicators; (**c**) Graph 3 displays the percentage values of eye movement indicators. The * in the figures indicates significant differences between R-G, R-R, and G-G for the corresponding indicators, with the color of the * matching the respective paradigm.

As shown in Figure 7, the eye movement data for the experimental group (R-G) and the control groups (R-R, G-G) were normalized across the three paradigms and displayed as line graphs with different colors representing each group.

Based on the data analysis results, six eye movement indicators showed significant differences between binocular color fusion and rivalry states. Specifically, Average Saccade Amplitude, Median Saccade Amplitude, and SD Saccade Amplitude exhibited significant differences across all tested paradigms. For instance, in the comparison between the R-G and R-R groups, the Average Saccade Amplitude values were 50.6% vs. 13.3%, 28.7% vs. 42.0%, and 54.1% vs. 14.0% across the three paradigms, respectively. These results indicate that saccade amplitude and its variability are stable indicators for distinguishing between binocular color fusion and rivalry, effectively reflecting the dynamic regulation strategies of the visual system under conflicting states.

Additionally, in the Gaze Stability paradigm, Average Blink Duration was significantly shorter in the R-G group compared to the R-R group (56.5% vs. 9.3%) and the G-G group (56.5% vs. 34.2%), suggesting higher visual attention and more pronounced blink suppression under this condition. In the Straight Curve Eye Hopping paradigm, Sac Avg Velocity (24.3% vs. 43.0%) and Sac Peak Velocity (42.9% vs. 28.4%) also showed significant differences between the R-G and G-G groups, indicating that during dynamic tasks, rivalry states may optimize conflict processing by modulating saccade velocity.

These findings collectively suggest that under binocular color rivalry states (R-G), the visual system enhances fixation stability and suppresses redundant eye movements to manage color conflicts, thereby achieving higher efficiency in visual information processing.

### 3.3. Results of Z-Score Normalization Analysis

Indicator values were subjected to a Z-Score normalization analysis, as shown in Table 1. In the present experiment, all eye movement data between the R-R group and the G-G group did not show significant differences. Therefore, in the Z-score normalization analysis, we combined these two groups into one. This allowed us to obtain the normalized range values for binocular color fusion and rivalry states across the three paradigms. The indicator ranges are expressed as normalized Mean ± SD.

In Table 1, a clear distinction can be made between binocular color fusion and rivalry based on the normalized ranges of eye movement indicators. For example, in the Gaze Stability paradigm, if the normalized value of Average Blink Duration falls within the range of −0.854–−0.454, it indicates that the subject is in the binocular color rivalry state. Conversely, when the normalized value lies within the range of −0.454–1.088, it suggests the subject is in the binocular color fusion state.

Similarly, in the Straight Curve Eye Hopping paradigm, eye movement indicators also exhibit distinct patterns for distinguishing between the two states. For instance, the normalized range of Average Saccade Amplitude in the binocular color rivalry state is −0.905–1.700, while in the fusion state, it is −1.919–−0.905. For Median Saccade Amplitude, the range in the rivalry state is −0.950–1.610, whereas in the fusion state, it is −1.980–−0.950. The SD Saccade Amplitude indicator shows a range of −0.827–1.545 in the rivalry state and −1.909–−0.827 in the fusion state. Additionally, Sac Avg Velocity exhibits a range of −1.404–0.376 in the rivalry state and 0.376–1.118 in the fusion state, while Sac Peak Velocity ranges from −0.676–2.308 in the rivalry state and −0.836–−0.684 in the fusion state.

In the Smoothed Eye Movement Tracking paradigm, similar distinguishing characteristics are observed. Specifically, the normalized range of Average Saccade Amplitude in the binocular color rivalry state is −1.392–0.608, compared to 0.608–2.234 in the fusion state. For Median Saccade Amplitude, the rivalry state range is −1.358–−0.552, while the fusion state range is −0.348–1.362. The SD Saccade Amplitude indicator shows a range of −1.196–−0.444 in the rivalry state and −0.444–1.498 in the fusion state.

In summary, the significant differences in the normalized ranges of eye movement indicators across different paradigms not only validate their effectiveness in distinguishing between binocular color fusion and rivalry but also provide quantifiable reference criteria for determining visual states based on eye movement features.

### 3.4. Results of Cross-Paradigm Merged Analysis

According to the cross-paradigm merged results of eye movement indicators shown in Table 2, Average Saccade Amplitude, Median Saccade Amplitude, and SD Saccade Amplitude exhibited significant differences across all three paradigms, demonstrating cross-paradigm stability. Specifically, when the range of Average Saccade Amplitude lies between 0.608 and 1.294, it can be inferred that the subject is in a state of binocular color fusion; conversely, when the range falls between −0.905 and −0.693, it indicates a state of binocular color rivalry. Similarly, for Median Saccade Amplitude, a range of −0.232–1.286 corresponds to binocular color fusion, while a range of −0.905–−0.693 suggests binocular color rivalry. Additionally, SD Saccade Amplitude indicates binocular color fusion when its range is −0.403–1.165, whereas a range of −0.827–−0.444 reflects binocular color rivalry. These findings demonstrate that even in less strictly controlled experimental states, eye movement indicators remain effective in distinguishing between states of binocular color fusion and rivalry.

### 3.5. Results of ROC Curve Analysis

The previous normalized analysis established specific thresholds for significant differences between binocular color fusion and rivalry. To further evaluate the sensitivity and specificity of these indicators in distinguishing the two visual phenomena, we conducted a ROC curve analysis. ROC curves, widely used in binary classification problems, plot the TPR on the vertical axis against the FPR on the horizontal axis [36].

This analysis enables us to visually compare and determine the discriminative efficacy of binocular color fusion and rivalry across different thresholds, allowing us to identify optimal thresholds that maximize accuracy and reliability in classifying color perception patterns. Consequently, we first calculated the sensitivity and specificity from the ROC curve and then derived Youden’s J statistic using the following Equations (1)–(3) [37]:

(1) J=Sensitivity+Specificity−1

(2) $$Sensitivity=\frac{TP}{TP+FN}\times 100\%$$

(3) $$Specificity=\frac{TN}{TN+FP}\times 100\%$$

Youden’s J statistic provides a comprehensive assessment of an eye movement indicator’s ability to recognize and discriminate between specific eye movement behaviors or mental processes in binocular rivalry. Sensitivity measures the proportion of trial periods during which the binocular rivalry phenomenon occurred and was correctly identified by the eye movement indicator. TP refers to instances where the model accurately detects binocular color rivalry (e.g., R-G) and indicates correct identifications of the absence of rivalry (e.g., binocular fusion, such as R-R and G-G). FP occurs when the model incorrectly identifies binocular color rivalry, and FN denotes instances where the presence of binocular rivalry is missed.

Specificity represents the proportion of trial periods without the phenomenon that were correctly classified by the eye movement indicator as free of significant rivalry dynamics. The true positive count reflects the number of samples the model correctly predicted as positive, whereas false negatives indicate samples inaccurately predicted as negative.

After calculating Youden’s J statistic, we plotted the ROC curve to visualize the classifier’s ability to distinguish between binocular color rivalry and binocular color fusion effectively.

In order to verify the independence of the data, we performed Spearman correlation analysis on the three eye movement indicators that showed significant differences, and the results showed that Average Saccade Amplitude, Median Saccade Amplitude, and SD Saccade Amplitude were significantly correlated with each other. For example, the correlation coefficient between Average Saccade Amplitude and Median Saccade Amplitude is greater than 0.9, as shown in Table 3.

**Figure 8:**
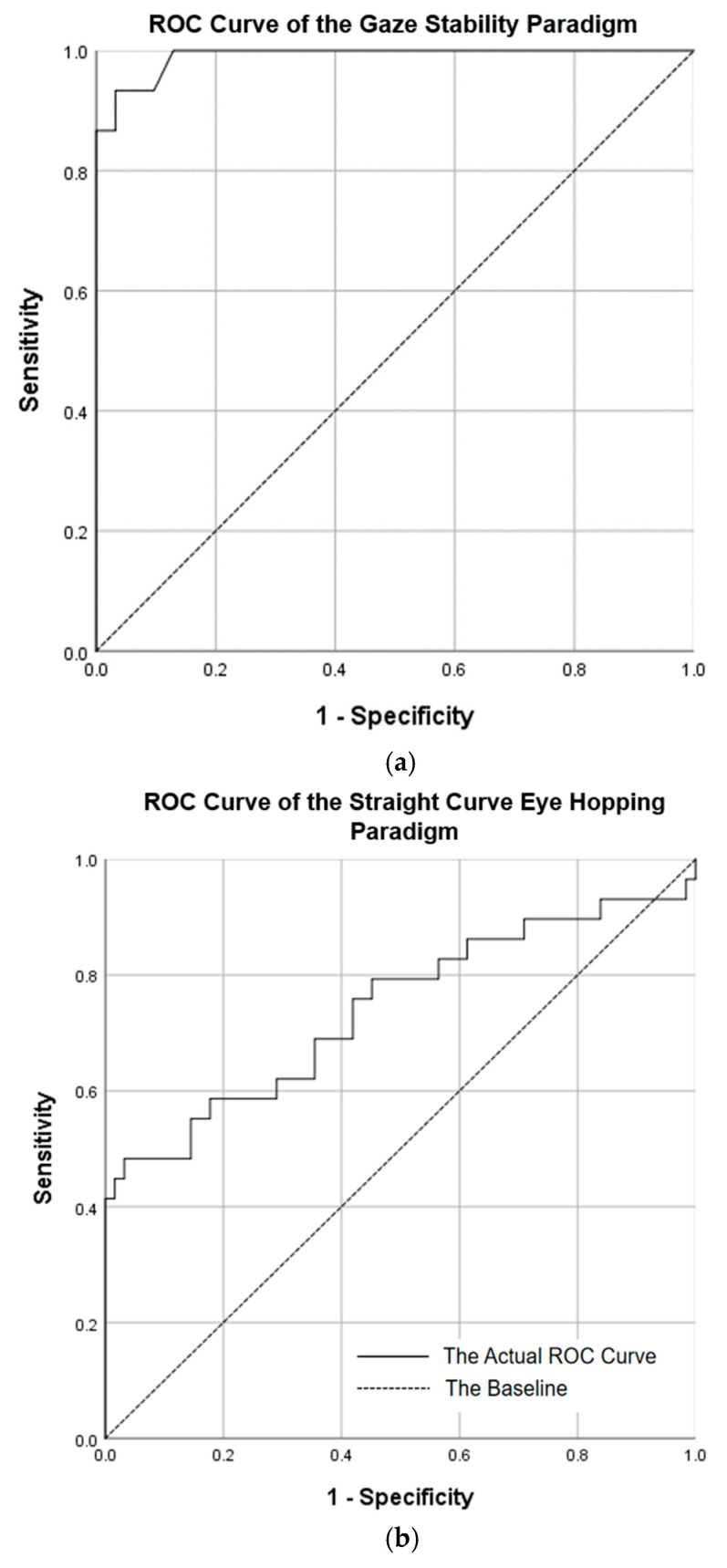

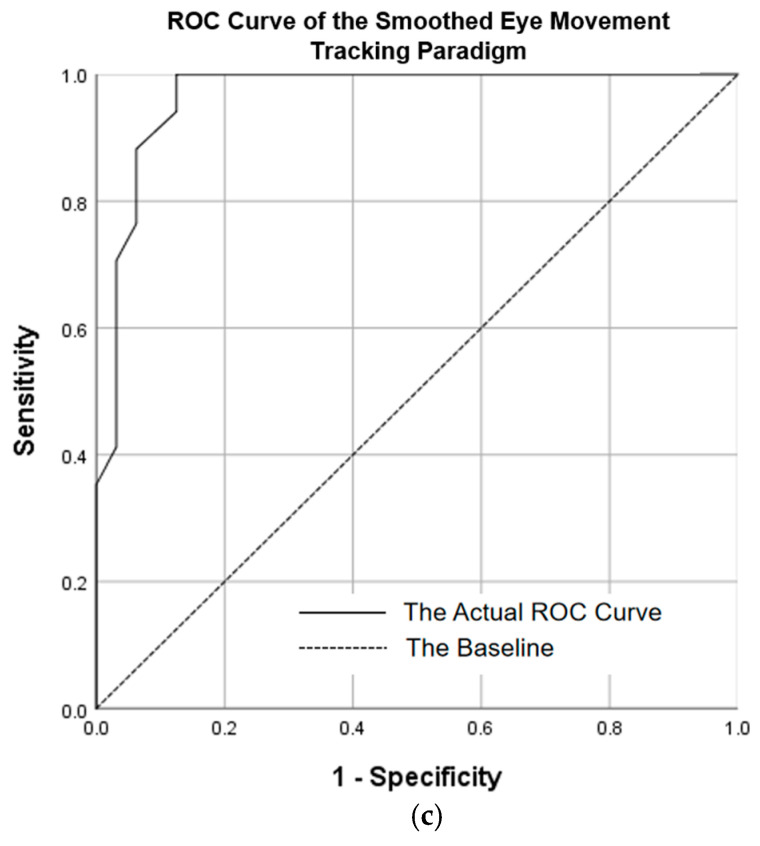
ROC curves for Different Eye Movement Paradigms. (**a**) ROC curve of the Gaze Stability paradigm. (**b**) ROC curve of the Straight Curve Eye Hopping paradigm. (**c**) ROC curve of the Smoothed Eye Movement Tracking paradigm. The actual ROC curve illustrates the relationship between sensitivity and specificity at various thresholds. The closer this curve approaches the top-left corner, the better the model’s performance. The baseline is the diagonal line representing the average value, with an AUC of 0.5.

In the Gaze Stability paradigm (Figure 8a), we selected four indicators with significant differences and excluded Average Blink Duration to avoid overfitting. The analysis revealed that Average Saccade Amplitude, Median Saccade Amplitude, and SD Saccade Amplitude effectively distinguished between binocular color fusion and rivalry, achieving an area under the ROC curve (AUC) of 0.990, with a sensitivity of 86.7% and specificity of 96.8%.

In the Straight Curve Eye Hopping paradigm (Figure 8b), we excluded SD Saccade Amplitude to simplify the model, resulting in an AUC of 0.741, a sensitivity of 86.2%, and a specificity of 61.3%. In the Smoothed Eye Movement Tracking paradigm (Figure 8c), which included all significant indicators, the AUC was 0.967, with a sensitivity of 70.6% and a specificity of 96.8%.

These results demonstrate the robust classification ability of the binary logistic regression model across the paradigms. The Gaze Stability paradigm models exhibited high accuracy in distinguishing between fusion and rivalry, while the Straight Curve Eye Hopping paradigm showed stable classification performance. The high AUC in the Smoothed Eye Movement Tracking paradigm further validates the effectiveness of these indicators in analyzing binocular color fusion and rivalry. Overall, the significant eye movement indicators in each paradigm are crucial for identifying binocular fusion and rivalry in various visual phenomena, underscoring the models’ reliable predictive and classification capabilities, which enhance our understanding of visual processing mechanisms.

## 4. Discussion

In this study, we conducted a systematic comparison of eye movement indicators under states of binocular color fusion (R-R group, G-G group) and rivalry (R-G group) through three paradigms: the Gaze Stability paradigm, Straight Curve Eye Hopping paradigm, and Smoothed Eye Movement Tracking paradigm. The findings reveal several critical points.

1. Differences in Eye Movement Indicators

Under normalization analysis, Saccade Amplitude-related indicators (Average Saccade Amplitude, Median Saccade Amplitude, SD Saccade Amplitude) showed significant differences across all three paradigms, indicating their robust response to visual conflict across tasks. This phenomenon is directly related to the core loop of conflict monitoring. For instance, during color rivalry states, the prefrontal–parietal network (PFC-PPC) dynamically adjusts saccade amplitude by modulating the color-selective gain of neurons in area V4 [38]. In the Gaze Stability paradigm, the Average Saccade Amplitude of the rivalry group was significantly lower than that of the fusion groups (rivalry group approximately 50.6%, R-R group 13.3%, G-G group 34.2%), likely reflecting how the lateral intraparietal area (LIP) maintains Gaze Stability by suppressing redundant saccades [39]. Conversely, in dynamic tasks, such as the Straight Curve Eye Hopping paradigm, the rivalry group’s Average Saccade Amplitude surpassed that of the fusion group (rivalry group 28.7%, fusion group 42.0%), suggesting that task demands drive the visual system to increase Saccade Amplitude for rapid attention shifts [40].

The reduction in SD Saccade Amplitude further supports this mechanism. For example, in the Gaze Stability paradigm, the SD for the rivalry group dropped to 54.1% (vs. R-R group at 14.0%), indicating that variability in the saccade strategy was actively suppressed. This suppression may be associated with phase synchronization of V4 γ oscillations (30–80 Hz), as Piantoni et al. found that phase locking of γ oscillations coordinates saccade initiation and perceptual switching, thereby reducing randomness [41]. Similarly, Denison et al. proposed that feedforward inhibition mechanisms optimize saccade planning by predicting conflicting inputs, thus reducing ineffective exploration [42].

Additionally, Average Blink Duration only showed significant differences in the Gaze Stability paradigm. The rivalry group’s blink duration (56.5%) was significantly shorter than that of the fusion groups (R-R group 9.3%, G-G group 34.2%), possibly due to the overactivation of the dorsal attention network (DAN). fMRI studies have shown that increased BOLD signals in the DAN correlate with blink suppression [43]. Color rivalry requires continuous engagement of the DAN to suppress input from the non-dominant eye, leading to compressed blink duration. In dynamic tasks, the priority of saccade speed diminishes the influence of the DAN on blink control, resulting in the disappearance of these differences.

For saccade velocity indicators (Sac Avg Velocity, Sac Peak Velocity), significant differences were observed in dynamic tasks, demonstrating adaptive strategies for conflict processing. In the Straight Curve Eye Hopping paradigm, the rivalry group’s Sac Avg Velocity and Sac Peak Velocity were significantly higher than those of the fusion group, illustrating a “speed-accuracy trade-off” strategy [44]. Specifically, burst firing of neurons in the Superior Colliculus (SC) increases speed by shortening saccade latency but may sacrifice spatial accuracy (e.g., an increase in endpoint error of about 5.2% in the rivalry group) [45]. Grossberg et al. further proposed that saccade velocity adjustment correlates with the prediction error of conflict signals; when the alternation frequency of color inputs increases, the SC accelerates saccades to shorten the decision window, matching the demands of dynamic inputs [46].

2. Difference Between Fusion and Rivalry States Based on Ranges

Our data further indicate that there are clear differences in the normalized ranges of various eye movement indicators between states of binocular color fusion and rivalry, confirming the effectiveness of these indicators in distinguishing the two visual states. For example, in the Gaze Stability paradigm, if the normalized value of Average Blink Duration falls within −0.854–−0.776, it can be preliminarily judged as rivalry, whereas if it falls within −0.455–1.406, it indicates fusion. The cross-paradigm merged analysis results show that Average Saccade Amplitude, Median Saccade Amplitude, and SD Saccade Amplitude effectively distinguish binocular color rivalry from fusion across all three paradigms. Specifically, when the range of Average Saccade Amplitude lies between 0.608 and 1.294, it suggests binocular color fusion; conversely, when it is between −0.905 and −0.693, it indicates rivalry. These results align with Blake and Logothetis’ dynamic threshold model, which successfully distinguishes visual rivalry from fusion using eye movement parameters based on statistical distribution differences [47]. Einhäuser et al. further validated the feasibility of multi-indicator classification methods, providing methodological support for constructing high-precision discrimination models in this study [48].

These indicators not only provide objective evidence for understanding binocular vision processing mechanisms but also hold broad practical application potential. By real-time monitoring of normalized eye movement indicator values, one can quickly differentiate between fusion and rivalry states, meeting the demand for immediate feedback in intelligent human–machine interaction systems. Additionally, specific eye movement indicator anomalies can aid in the early detection of visual conflict processing abnormalities, offering auxiliary information for early screening and clinical diagnosis of visual disorders. Furthermore, quantitative data provide solid experimental evidence for building and validating binocular vision neural computation models, advancing our understanding of visual information integration mechanisms.

3. ROC Analysis and Classification Efficacy

Further validation through ROC curve analysis demonstrated the effectiveness of the three eye movement paradigms in distinguishing between states of binocular color fusion and rivalry. The Gaze Stability paradigm exhibited nearly perfect classification efficacy (AUC = 0.990), with high specificity (96.8%) and substantial sensitivity (86.7%), significantly outperforming certain neuro-psychiatric disorder diagnostic studies based on MRI biomarkers, such as those reported by Kambeitz et al. with a sensitivity of 77% and a specificity of 78% [49]. This not only highlights the potential of eye movement indicators in visual state classification but also showcases their unique advantages in high-precision visual state assessment.

Although the Straight Curve Eye Hopping paradigm had a lower AUC (0.741), its screening potential at high specificity ranges (>80%, with a sensitivity of 62.1%) indicates that this paradigm can serve as an initial screening tool. In contrast, the Smoothed Eye Movement Tracking paradigm displayed high classification efficacy (AUC = 0.967) and specificity (96.8%), albeit with some variation in sensitivity (70.6%), possibly reflecting biological variability among subjects in feedforward prediction mechanisms [50]. Notably, the ROC curves for all three paradigms exhibited bimodal distributions, supporting the theoretical hypothesis of discrete stable states for color fusion and rivalry. The performance gradient across paradigms (Gaze Stability paradigm > Smoothed Eye Movement Tracking paradigm > Straight Curve Eye Hopping paradigm) further maps onto the neural hierarchy of visual processing; static maintenance (high-level cognitive regulation) surpasses dynamic tracking (mid-level motor integration) and path planning (low-level reflex pathways).

These findings not only confirm the classification validity of eye movement indicators but also establish a multi-paradigm joint analysis framework. Future research should incorporate time-resolved ROC analyses and include spatial features, such as saccade trajectory curvature, to enhance model interpretability.

Overall, this study, through quantitative evaluation and multi-paradigm experimental validation, further demonstrates the effectiveness of eye movement indicators in distinguishing between binocular color fusion and rivalry states. It provides robust theoretical and methodological support for the fine assessment of visual states and related applications, such as real-time monitoring, visual disorder screening, and the construction of neural computation models.

## 5. Conclusions

Color fusion and rivalry are two key mechanisms of information integration in binocular vision, characterizing how the visual system processes consistent and conflicting inputs, respectively. This study systematically revealed the quantitative differences in eye movement indicators under states of binocular color fusion and rivalry through multi-paradigm eye movement experiments, validating the sensitivity of indicators such as Saccade Amplitude and velocity to visual conflict.

These eye movement indicators not only effectively distinguish between states of binocular color fusion and rivalry but also show significant characteristic changes across different visual processing modes. Through Z-Score analysis, we found that eye movement indicators could effectively discriminate between binocular color fusion and rivalry states. Furthermore, ROC curve analysis further confirmed the effectiveness of the experimental paradigms employed—including the Gaze Stability paradigm, Straight Curve Eye Hopping paradigm, and Smoothed Eye Movement Tracking paradigm—in elucidating the mechanisms of binocular color fusion and rivalry. These results not only validate the sensitivity of eye movement indicators to visual conflict but also highlight their potential as biomarkers for characterizing dynamic visual processing. Additionally, our findings suggest that eye movement behavior can serve as a biomarker for characterizing the dynamic processing of visual conflict, providing empirical support for understanding the neural computational models of binocular vision.

## Figures and Tables

**Table 1:** Z-score normalized analysis of eye movement indicators with significant differences. Mean ± SD represents the corresponding values of Mean ± SD under Z-Score normalization analysis, while Range indicates the range of the indicator corresponding to these values.

Paradigm	Indicators	Binocular Color Fusion (*n* = 36)		Binocular Color Rivalry (*n* = 18)	
		Mean ± SD	Range	Mean ± SD	Range
Gaze Stability	Average Blink Duration	0.317 ± 0.771	−0.454~1.088	−0.654 ± 0.2	−0.854~−0.454
	Average Saccade Amplitude	0.525 ± 0.769	−0.244~1.294	−1.082 ± 0.389	−1.471~−0.693
	Median Saccade Amplitude	0.527 ± 0.759	−0.232~1.286	−1.088 ± 0.405	−1.493~−0.683
	SD Saccade Amplitude	0.381 ± 0.784	−0.403~1.165	−0.785 ± 0.382	−1.167~−0.403
Straight Curve Eye Hopping	Average Saccade Amplitude	−1.412 ± 0.507	−1.919~−0.905	0.397 ± 1.302	−0.905~1.700
	Median Saccade Amplitude	−1.455 ± 0.525	−1.980~−0.950	0.33 ± 1.28	−0.950~1.610
	SD Saccade Amplitude	−1.368 ± 0.541	−1.909~−0.827	0.359 ± 1.186	−0.827~1.545
	Sac Avg Velocity	0.747 ± 0.371	0.376~1.118	−0.509 ± 0.895	−1.404~0.376
	Sac Peak Velocity	−0.760 ± 0.076	−0.836~−0.684	0.816 ± 1.492	−0.676~2.308
Smoothed Eye Movement Tracking	Average Saccade Amplitude	1.421 ± 0.813	0.608~2.234	−1 ± 0.392	−1.392~0.608
	Median Saccade Amplitude	0.507 ± 0.855	−0.348~1.362	−0.955 ± 0.403	−1.358~−0.552
	SD Saccade Amplitude	0.527 ± 0.971	−0.444~1.498	−0.82 ± 0.376	−1.196~−0.444

**Table 2:** Cross-paradigm merged analysis of eye movement indicators with significant differences. Mean ± SD represents the corresponding values of Mean ± SD under cross-paradigm merged analysis, while Range indicates the range of the indicator corresponding to these values.

Indicators	Binocular Color Fusion (*n* = 36)		Binocular Color Rivalry (*n* = 18)	
	Mean ± SD	Range	Mean ± SD	Range
Average Saccade Amplitude	0.951 ± 0.343	0.608~1.294	−0.799 ± 0.106	−0.905~−0.693
Median Saccade Amplitude	0.527 ± 0.759	−0.232~1.286	−0.817 ± 0.134	−0.950~−0.683
SD Saccade Amplitude	0.381 ± 0.784	−0.403~1.165	−0.636 ± 0.192	−0.827~−0.444

**Table 3:** Correlation analysis of eye movement indicators in the Gaze Stability paradigm group.

Indicators	Average Saccade Amplitude	Median Saccade Amplitude	SD Saccade Amplitude
Average Saccade Amplitude	1 (0.000)	0.95 (0.000)	0.54 (0.000)
Median Saccade Amplitude	0.95 (0.000)	1 (0.000)	0.52 (0.000)
SD Saccade Amplitude	0.54 (0.000)	0.52 (0.000)	1 (0.000)

**Figure A1:**
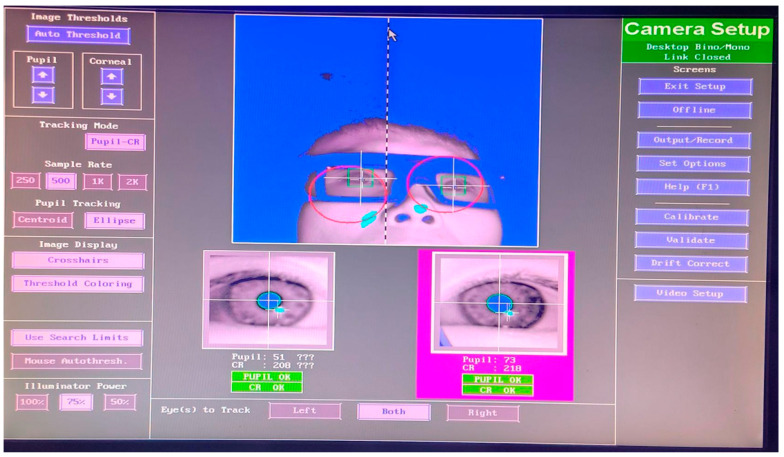
Calibration process of eye locking.

**Figure A2:**
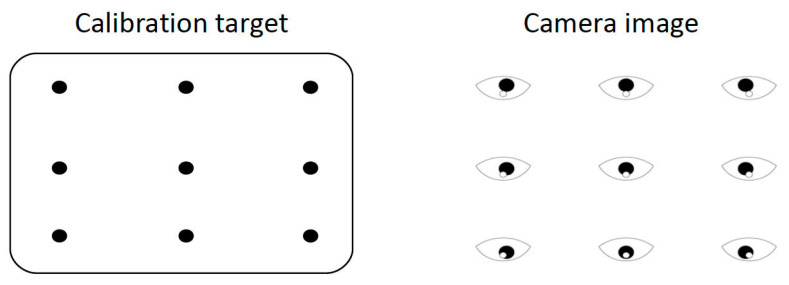
Nine-point calibration diagram.

## Data Availability

The original contributions presented in the study are included in the article, and stimuli and raw data are available at https://doi.org/10.6084/m9.figshare.25688625.v1 (dataset accessed on 25 April 2024).

## Appendix A

**Table A1:** Data of acuity optometric screening.

Total Participants (Non-Myopes/Myopes)	Non-Myopes	Myopes			Stereoacuity@41cm (Arcsec)
	Naked Visual Acuity @4m	Naked Eye Acuity@4m	Corrective Acuity@4m	Corrected Degree(D)	
18(11/7)	OS:20/20 OD:20/20	OS:20/40~20/25	OS:20/20	OS: −1.75 ~−6.00	41~55
		OD:20/40~20/25	OD:20/20	OD: −2.75~−6.00	

**Table A2:** Analysis of interocular differences (stability gaze, bold part indicates significant difference).

Indicators	Name	Mean	SD	t	*p*	Cohen’s d
Acceleration X	Left	−10.235	1150.613	0.081	0.935	0.004
	Right	−44.346	1233.721			
Acceleration Y	Left	9.048	520.388	0.337	0.736	0.015
	Right	−32.468	3865.833			
Gaze X	Left	251.948	78.303	37.77	**0.000**	1.689
	Right	60.968	139.41			
Gaze Y	Left	377.523	5.743	107.402	**0.000**	4.803
	Right	787.018	120.432			
Pupil Size	Left	3799.668	839.031	9.425	**0.000**	0.422
	Right	3447.723	830.9			
Velocity X	Left	0.35	7.997	0.136	0.892	0.006
	Right	0.178	39.272			
Velocity Y	Left	0.363	3.82	1.225	0.221	0.055
	Right	−0.131	12.183			

**Table A3:** Analysis of interocular differences (Straight Curve Eye Hopping, bolded text indicates significant differences).

Indicators	Name	Mean	SD	t	*p*	Cohen’s d
Acceleration X	Left	−21.48	1268.941	−0.545	0.586	0.023
	Right	37.983	3387.998			
Acceleration Y	Left	−4.973	1272.723	0.895	0.371	0.038
	Right	−112.16	3761.714			
Gaze X	Left	499.159	142.151	−0.515	0.606	0.022
	Right	502.14	128.92			
Gaze Y	Left	396.257	127.343	−4.794	**0.000**	0.204
	Right	421.58	120.324			
Pupil Size	Left	1597.771	801.02	7.565	**0.000**	0.323
	Right	1378.965	527.798			
Velocity X	Left	−0.19	18.995	−0.448	0.654	0.019
	Right	0.226	24.279			
Velocity Y	Left	1.33	11.553	0.509	0.611	0.022
	Right	0.989	19.003			

**Table A4:** Analysis of interocular differences (Smoothed Eye Movement Tracking, bold parts indicate significant differences).

Indicators	Eye	Mean	SD	t	*p*	Cohen’s d
Acceleration X	Left	14.163	497.723	0.533	0.594	0.028
	Right	−6.692	1056.822			
Acceleration Y	Left	−29.448	864.872	−0.125	0.9	0.006
	Right	−19.085	2365.992			
Gaze X	Left	408.603	73.545	−12.566	**0.000**	0.65
	Right	453.215	59.399			
Gaze Y	Left	487.712	55.7	−1.94	0.053	0.1
	Right	493.012	47.51			
Pupil Size	Left	2725.338	672.047	22.001	**0.000**	1.138
	Right	1980.695	622.701			
Velocity X	Left	0.388	3.645	−0.339	0.735	0.018
	Right	0.455	4.094			
Velocity Y	Left	0.324	7.426	−0.04	0.968	0.002
	Right	0.342	10.539			

**Table A5:** Normal distribution test table for eye movement indicators in different paradigms (parentheses represent *p*-values; bolded portions represent significant differences).

Indicators	Gaze Stability (*n* = 54)			Smoothed Eye Movement Tracking (*n* = 108)			Straight Curve Eye Hopping (*n* = 54)		
	Skewness	Kurtosis	S-W Test	Skewness	Kurtosis	S-W Test	Skewness	Kurtosis	S-W Test
Average Blink Duration	0.92	−0.61	0.78 **(0.000)**	1.88	2.25	0.59 **(0.000)**	2.67	6.21	0.45 **(0.000)**
Average Fixation Duration	0.54	−0.31	0.95 **(0.036)**	2.16	8.21	0.82 **(0.000)**	0.39	0.19	0.98 (0.439)
Average Saccade Amplitude	−0.02	−1.19	0.96 **(0.047)**	2.96	11.22	0.69 **(0.000)**	0.19	1.04	0.96 **(0.083)**
Blink Count	0.92	0.66	0.77 **(0.000)**	2.37	6.76	0.59 **(0.000)**	2.80	7.46	0.45 **(0.000)**
Fixation Count	0.85	0.36	0.85 **(0.000)**	1.14	0.97	0.82 **(0.000)**	0.53	0.27	0.88 **(0.000)**
Median Fixation Duration	0.38	−0.52	0.97 (0.181)	2.03	7.14	0.83 **(0.000)**	0.28	0.45	0.99 (0.765)
Median Saccade Amplitude	0.001	−1.14	0.96 (0.079)	3.10	11.98	0.67 **(0.000)**	0.14	1.12	0.96 (0.054)
SD Saccade Amplitude	0.39	−1.09	0.93 **(0.004)**	2.17	5.59	0.77 **(0.000)**	0.33	1.31	0.92 **(0.001)**
SD Fixation Duration	1.19	1.60	0.90 **(0.000)**	1.93	4.38	0.80 **(0.000)**	0.18	0.26	0.95 **(0.035)**
Saccade Count	0.59	0.38	0.87 **(0.000)**	0.57	−0.03	0.88 **(0.000)**	0.04	0.67	0.85 **(0.000)**
Pupil Size Duration	−0.60	0.65	0.97 (0.152)	0.52	1.43	0.95 **(0.001)**	1.00	2.79	0.94 **(0.009)**
Sac Avg Velocity	1.11	1.62	0.91 **(0.001)**	0.01	−1.21	0.95 **(0.001)**	1.15	2.97	0.93 **(0.003)**
Sac Peak Velocity	1.47	1.66	0.82 **(0.000)**	2.28	4.07	0.52 **(0.000)**	2.95	9.80	0.64 **(0.000)**

**Table A6:** Results of the independent sample *t*-test for the experimental group and the control group (stability gaze, df = 34).

Indicators	Exp./Ctr.	Mean	SD	*p*	Cohen’s d
Median Fixation Duration (ms)	R-G/R-R	244.9/254.1	105.7/135.1	0.823	0.1
	R-G/G-G	244.9/220.7	105.7/101.2	0.547	0.2
	R-R/G-G	254.1/220.7	135.1/101.2	0.408	0.3
Median Saccade Amplitude (°)	R-G/R-R	9.4/23.1	5.1/7.1	0.000	0.5
	R-G/G-G	9.4/36.4	5.1/6.9	0.000	0.5
	R-R/G-G	23.1/36.4	7.1/6.9	0.000	0.1
Pupil Size Duration (ms)	R-G/R-R	4773.0/4479.9	1418.3/767.2	0.476	0.3
	R-G/G-G	4773.0/4439.9	1418.3/1180.1	0.412	0.3
	R-R/G-G	4479.9/4439.9	767.2/1180.1	0.921	0.0

**Table A7:** Results of the independent sample Mann–Whitney U test for binocular color fusion and rivalry (stability gaze, bold indicates significant difference).

Indicators	Exp./Ctr.	Mean	SD	*p*	Cohen’s d
Average Blink Duration (ms)	R-G/R-R	1.0/9.5	1.6/6.3	0.002	1.451
	R-G/G-G	1.0/19.5	1.6/11.5	0.009	1.427
	R-R/G-G	9.5/19.5	6.3/11.5	0.145	0.546
Average Fixation Duration (ms)	R-G/R-R	199.4/260.5	120.5/118.8	0.752	0.155
	R-G/G-G	199.4/243.0	120.5/114.7	0.669	0.071
	R-R/G-G	260.5/243.0	118.8/114.7	0.874	0.087
Average Saccade Amplitude (°)	R-G/R-R	9.5/26.5	5.3/6.8	0.000	2.681
	R-G/G-G	9.5/38.5	5.3/10.4	0.000	3.230
	R-R/G-G	26.5/38.5	6.8/10.4	0.000	1.167
Blink Count (times)	R-G/R-R	0.0/1.0	0.6/0.9	0.035	0.739
	R-G/G-G	0.0/1.0	0.6/0.6	0.128	0.462
	R-R/G-G	1.0/1.0	0.9/0.6	0.375	0.372
Fixation Count (times)	R-G/R-R	2.0/2.0	1.2/0.9	0.495	0.101
	R-G/G-G	2.0/3.0	1.2/1.1	0.261	0.281
	R-R/G-G	2.0/3.0	0.9/1.1	0.477	0.216
SD Saccade Amplitude (°)	R-G/R-R	6.5/19.5	4.1/7.8	0.000	1.662
	R-G/G-G	6.5/30.5	4.1/13.1	0.001	1.760
	R-R/G-G	19.5/30.5	7.8/13.1	0.038	0.629
SD Fixation Duration (ms)	R-G/R-R	79.2/89.9	100.7/88.7	0.183	0.331
	R-G/G-G	79.2/90.7	100.7/71.3	0.516	**0.044**
	R-R/G-G	89.9/90.7	88.7/71.3	0.429	0.343
Saccade Count (times)	R-G/R-R	2.0/2.0	1.2/0.8	0.402	0.159
	R-G/G-G	2.0/2.0	1.2/0.9	0.227	0.306
	R-R/G-G	2.0/2.0	0.8/0.9	0.564	0.190
Sac Avg Velocity (°/s)	R-G/R-R	59.3/79.4	28.8/46.7	0.402	0.435
	R-G/G-G	59.3/78.2	28.8/24.0	0.669	0.088
	R-R/G-G	79.4/78.2	46.7/24.0	0.591	0.391
Sac Peak Velocity (°/s)	R-G/R-R	139.4/126.8	177.3/183.1	0.862	0.180
	R-G/G-G	139.4/187.9	177.3/213.1	0.150	0.418
	R-R/G-G	126.8/187.9	183.1/213.1	0.393	0.249

**Table A8:** Results of the independent sample *t*-test for the experimental group and the control group (*n* = 36, Straight Curve Eye Hopping, bold text indicates a significant difference).

Independent Samples *t*-Test Result (df = 34)						
Indicators	Exp./Ctr.	Average	Standard	*p*	t	Cohen’s d
Average Fixation Duration (ms)	R-G/R-R	260.6/283.1	83.8/94.2	0.461	−0.7	0.3
	R-G/G-G	260.6/265.7	83.8/86.2	0.870	−0.2	0.1
	R-R/G-G	283.1/265.7	94.2/86.2	0.581	−9.6	3.4
Average Saccade Amplitude (°)	R-G/R-R	9.5/21.6	4.9/6.0	**0.000**	−6.4	2.2
	R-G/G-G	9.5/36.7	4.9/7.8	**0.000**	−9.4	3.3
	R-R/G-G	21.6/36.7	6.0/7.8	**0.000**	−0.1	0.0
Median Fixation Duration (ms)	R-G/R-R	251.9/280.9	98.6/100.4	0.390	−0.8	0.3
	R-G/G-G	251.9/263.5	98.6/91.9	0.738	−0.3	0.1
	R-R/G-G	280.9/263.5	100.4/91.9	0.617	0.5	0.2
Median Saccade Amplitude (°)	R-G/R-R	9.8/21.5	5.3/8.0	**0.000**	−6.2	2.1
	R-G/G-G	9.8/37.7	5.3/7.8	**0.000**	−9.4	3.3
	R-R/G-G	21.5/37.7	8.0/7.8	**0.000**	−0.2	0.1

**Table A9:** Results of the independent sample Mann–Whitney U test for the experimental and control groups (*n* = 36, Straight Curve Eye Hopping,).

Independent Sample Mann–Whitney U Test Result					
Indicators	Exp./Ctr.	Median	Standard Deviation	*p*	Cohen’s d
Average Blink Duration (ms)	R-G/R-R	1.0/1.0	0.5/1.6	0.428	0.528
	R-G/G-G	1.0/1.0	0.5/3.3	0.284	0.644
	R-R/G-G	1.0/1.0	1.6/3.3	0.492	0.391
Blink Count (times)	R-G/R-R	0.0/0.0	0.8/0.4	0.172	0.752
	R-G/G-G	0.0/0.0	0.8/0.9	0.435	0.133
	R-R/G-G	0.0/0.0	0.4/0.9	0.582	0.337
Fixation Count (times)	R-G/R-R	2.5/2.0	1.2/0.7	0.349	0.403
	R-G/G-G	2.5/2.0	1.2/0.8	0.331	0.391
	R-R/G-G	2.0/2.0	0.7/0.8	0.875	0.000
SD Saccade Amplitude (°)	R-G/R-R	8.5/20.5	4.5/8.3	0.001	1.493
	R-G/G-G	8.5/33.5	4.5/12.4	0.000	2.203
	R-R/G-G	20.5/33.5	8.3/12.4	0.002	0.996
SD Fixation Duration (ms)	R-G/R-R	102.5/129.9	61.4/75.6	0.168	0.489
	R-G/G-G	102.5/151.3	61.4/76.0	0.071	0.588
	R-R/G-G	129.9/151.3	75.6/76.0	0.874	0.092
Saccade Count (times)	R-G/R-R	2.0/2.0	1.0/0.7	0.683	0.127
	R-G/G-G	2.0/2.0	1.0/0.5	0.345	0.273
	R-R/G-G	2.0/2.0	0.7/0.5	0.548	0.177
Pupil Size Duration (ms)	R-G/R-R	3889.5/3843.5	1050.6/1334.5	0.591	0.22
	R-G/G-G	3889.5/3929.0	1050.6/1574.2	0.569	0.335
	R-R/G-G	3843.5/3929.0	1334.5/1574.2	0.849	0.126
Sac Avg Velocity (°/s)	R-G/R-R	53.4/53.6	12.7/15.6	0.569	0.254
	R-G/G-G	53.4/53.6	12.7/24.1	0.975	0.144
	R-R/G-G	53.6/53.6	15.6/24.1	0.704	0.041
Sac Peak Velocity (°/s)	R-G/R-R	71.8/82.0	31.0/75.3	0.411	0.36
	R-G/G-G	71.8/68.6	31.0/69.0	0.874	0.263

## Appendix B. Eye Movement Instrument Calibration Process

The eye tracker was accurately calibrated prior to the experiment and is only described in Section 2.4 of the text for fear of being too large. Before the experiment officially began, subjects were required to put on their active stereo glasses, adjust their sitting position, and perform a nine-point calibration of the eye tracker to enable it to capture the subject’s eye movements well. The calibration is reported in detail below:

(1) Subjects completed an informed consent form describing the general procedure.
(2) Turn off or mute all distracting electronic devices.
(3) Turn on the power supply, power on the host PC and display PC, and wait for the devices to display the interface normally.
(4) Adjust the subject’s seat height so that they can comfortably rest their chin on the chin rest and lean their forehead against the forehead. Locate the captured eye roughly in the center of the display screen or 1/4 of the way from the upper screen.
(5) On the host PC, click the red circle on the subject’s eye to lock the tracking range, press A to automatically adjust the threshold, and adjust the lens focus so that the eye is clearly visible.
(6) Have the subject view the four corners of the monitor, making sure that the pupillary and corneal reflections are tracked over the entire surface of the monitor. If the pupillary or corneal reflexes are lost at the edges of the monitor, make sure the screen is far enough away from the subject. For subjects wearing eyeglasses, the frame sometimes obscured part of the video image of the eye when viewing extreme horizontal or vertical angles, which was adjusted by moving the front of the subject’s chin.
(7) Calibration is the process used to set up the eye tracking software to accurately track eye movements; in this experiment, a nine-point calibration was used in order to optimize the calibration accuracy.
(8) Confirm that the subject’s eye signal is intact and click the Calibrate button on the host PC interface to begin calibration. During calibration, instruct the subject to look at each point until it disappears and then look at the next point.
(9) Validate the calibration. Click the Validate button to confirm the calibration and decide whether it is acceptable or not based on the feedback.
(10) Start the experiment after an acceptable calibration. Click the Output/Record button to begin presenting the stimulus and recording data. Instruct the subject not to talk while the stimulus is displayed. Talking will cause the head to move up and down at the chin, which will decrease the accuracy of eye tracking.
(11) If the subject needs to rest at any time or the quality of the eye movement data decreases, the calibration will be checked and recalibrated as needed.
(12) Upon completion of the experiment, check for the presence of eye movement data in the data folder.
(13) Ask the subject if any discomfort exists, and if not, have the subject leave the laboratory.
